# A retrospective cohort study of factors relating to the longitudinal change in birth weight

**DOI:** 10.1186/s12884-015-0777-8

**Published:** 2015-12-22

**Authors:** Kelly S. Gibson, Thaddeus P. Waters, Douglas D. Gunzler, Patrick M. Catalano

**Affiliations:** Department of Obstetrics and Gynecology, MetroHealth Medical Center, Case Western Reserve University School of Medicine, 2500 Metrohealth Drive, Cleveland, OH 44109 USA; Loyola University Medical Center, 2160 S 1st Ave, Maywood, IL 60153 USA; Center for Health Care Research & Policy, Case Western Reserve University School of Medicine, 2500 Metrohealth Drive, Cleveland, OH 44109 USA

**Keywords:** Birth weight, Birth length, Ponderal index

## Abstract

**Background:**

Recent reports have shown a decrease in birth weight, a change from prior steady increases. Therefore we sought to describe the demographic and anthropometric changes in singleton term fetal growth.

**Methods:**

This was a retrospective cohort analysis of term singleton deliveries (37–42 weeks) from January 1, 1995 to January 1, 2010 at a single tertiary obstetric unit. We included all 43,217 neonates from term, singleton, non-anomalous pregnancies. Data were grouped into five 3-year intervals. Mean and median birth weight (BW), birth length (BL), and Ponderal Index (PI) were estimated by year, race and gestational age. Our primary outcome was change in BW over time. The secondary outcomes were changes in BL and PI over time.

**Results:**

Mean and median BW decreased by 72 and 70 g respectively (*p* < 0.0001) over the 15 year period while BL also significantly decreased by 1.0 cm (*P* < 0.001). This contributed to an increase in the neonatal PI by 0.11 kg/m^3^ (*P* < 0.001). Mean gestational age at delivery decreased while maternal BMI at delivery, hypertension, diabetes, and African American race increased. Adjusting for gestational age, race, infant sex, maternal BMI, smoking, diabetes, hypertension, and parity, year of birth contributed 0.1 % to the variance (−1.7 g/year; 26 g) of BW, 1.8 % (−0.06 cm/year; 0.9 cm) of BL, and 0.7 % (+0.008 kg/m^3^/year; 0.12 kg/m^3^) of PI. These findings were independent of the proportional change in race or gestational age.

**Conclusions:**

We observed a crude decrease in mean BW of 72 g and BL of 1 cm over 15 years. Furthermore, once controlling for gestational age, race, infant sex, maternal BMI, smoking, diabetes, hypertension, and parity, we identified that increasing year of birth was associated with a decrease in BW of 1.7 g/year. The significant increase in PI, despite the decrease in BW emphasizes the limitation of using birth weight alone to define changes in fetal growth.

**Electronic supplementary material:**

The online version of this article (doi:10.1186/s12884-015-0777-8) contains supplementary material, which is available to authorized users.

## Background

For decades, newborn birth weight has been a focus of clinical investigation as an assessment of fetal growth. During the 1990s several investigators reported a steady increase in newborn birth weight. [1–9] However, more recent investigations have suggested a plateauing [10–12] or a possible reversal in this trend [13–15]. These observations have raised several questions regarding fetal development because of possible long-term sequellae. As fetal growth (both excessive and restricted) is associated with adverse long-term health, further evaluation of these observations is warranted.

The intrauterine environment, genetics, and gestational age all contribute to newborn weight. Maternal factors including hypertensive disorders [16–18], diabetes [19], smoking [20, 21], body mass index (BMI) [22], gestational weight gain [23], and race [24] play key roles in the complex process of fetal growth [25]. The frequency of these variables within the population has not remained constant. Therefore, to more accurately evaluate the changes in birth weight over time, one should account for population changes such as the increase in obesity in women of reproductive age [26], comorbid conditions, and changes in obstetric practice [27, 28]. Additionally, while recent observational studies have noted a decrease in term birth weight, these data are incongruent with the corresponding increase or plateauing in childhood obesity [29, 30].

Therefore, the primary aim of this study was to evaluate the change in term singleton newborn birth weight over 15 years at a tertiary inner city hospital, while adjusting for relevant factors that contribute to fetal growth. As a secondary aim, we evaluated the changes in Ponderal Index (an anthropometric estimate of neonatal adiposity analogous to a BMI in adults) during this time period to better estimate the changes in weight for length as a measure of neonatal adiposity.

## Methods

A contemporaneously maintained database was used to examine the impact of maternal demographic characteristics and co-morbid conditions upon fetal growth among term singleton deliveries. In brief, delivery records from the Department of Obstetrics and Gynecology, MetroHealth Medical Center, Cleveland, Ohio, are entered prospectively into a computerized database for research purposes. The data collection began in October 1974, and data such as maternal history, pregnancy complications, and delivery details are ascertained for each patient. Each entry is independently compared with the patient’s medical record by an independent reviewer [31]. Only data collected from January 1995 to January 2010 were used as this extended our prior report, which showed an increase in our population’s birth weight [31]. This study was reviewed and approved by The MetroHealth System Institutional Review Board. As the study was on de-identified data from a departmental database, it received a waiver of HIPPA and informed consent under an expedited review.

Of the 49,550 singleton non-anomalous live born births recorded between January 1, 1995 and January 1, 2010, 43,217 were included in the analysis. We excluded births with gestational age less than 37 weeks (6287) or greater than 42 completed weeks (38) and records with a birth weight that was inconsistent with gestational age (greater than 2.5 standard deviations) based on the data for United States term birth weight by Alexander et al. [32].

Gestational age was defined by best obstetrical estimate, normally including the patient’s last menstrual period with ultrasound confirmation usually less than 20 weeks gestation. In cases where there was a discrepancy, we used the estimated gestational age calculated by ultrasound dating. We queried data on variables relevant to newborn birth weight including maternal age, parity, self-identified race (Caucasian, African-American, Hispanic, Asian, Other, and Declined/no answer), smoking, illicit drug use, prenatal care, weight at delivery, height, complications of pregnancies (hypertensive disorders, diabetes, and bleeding), and mode of delivery. To assess fetal growth, infant birth weight, birth length, weight for length as an estimate of adiposity, and sex were abstracted. Birth weight (BW) was measured at delivery on a scale that is calibrated monthly. Birth length (BL) is measured from the crown of the newborn’s head in a neutral position to the heel of the fully extended leg using a disposable tape measure. These methods have been used consistently over the time of our review by our nursing staff. Additionally, we estimated the change in newborn Ponderal Index (PI: calculated as kg/m^3^) over time as a marker of fetal weight for length an estimate of adiposity [33].

The change in newborn BW, BL and PI in addition to changes in patient demographics and medical comorbidities were analyzed as 3 year intervals to examine trends over time. We used the Cochran-Armitage test for trend for the binary variables, nulliparity, smoking, cesarean delivery, hypertension, diabetes mellitus, and infant sex. We used regression analysis with contrasts for testing a linear trend across the 3 year intervals for the continuous variables (maternal age, BMI, percent African American, mean and median BW, gestational age, BL, and PI). We also described the total change from 1995 to 2010 of each of these variables. Additionally, the changes in mean and median BW were estimated by year, race and gestational age.

We initially evaluated if the changes in BW, BL and PI (dependent variables) over time were correlated with the changes in all available independent variables (maternal age, parity, BMI at delivery, race, smoking, diabetes mellitus, and hypertension and neonatal gestational age) in univariate correlation analyses (Additional file 1: Table S1). We then included all the significant and clinically relevant independent variables into multiple regression models for each outcome (BW, BL and PI). These multivariate models were used to determine the contribution of each independent variable, adjusted for the potential confounding of other included variables, with BW BL and PI, respectively. We tested for multi-colinearity by examining the correlation between predictors (Additional file 1: Table S2). The covariates for race, parity, infant sex, smoking, diabetes, and hypertension were treated as binary variables in our final models. Further, the interaction between race and sex was explored in the analyses. Separate regressions were performed for each race and compared with the Chow test.

A *p* value <0.05 was considered significant. All statistical analysis was done using Statview 5.01 (Cary, NC) and SAS Version 9.2 (Cary, NC SAS Institute Inc). Due to the large sample size in our study, we particularly evaluated the effect size as an indicator of magnitude of each relationship.

## Results

**Fig. 1:**
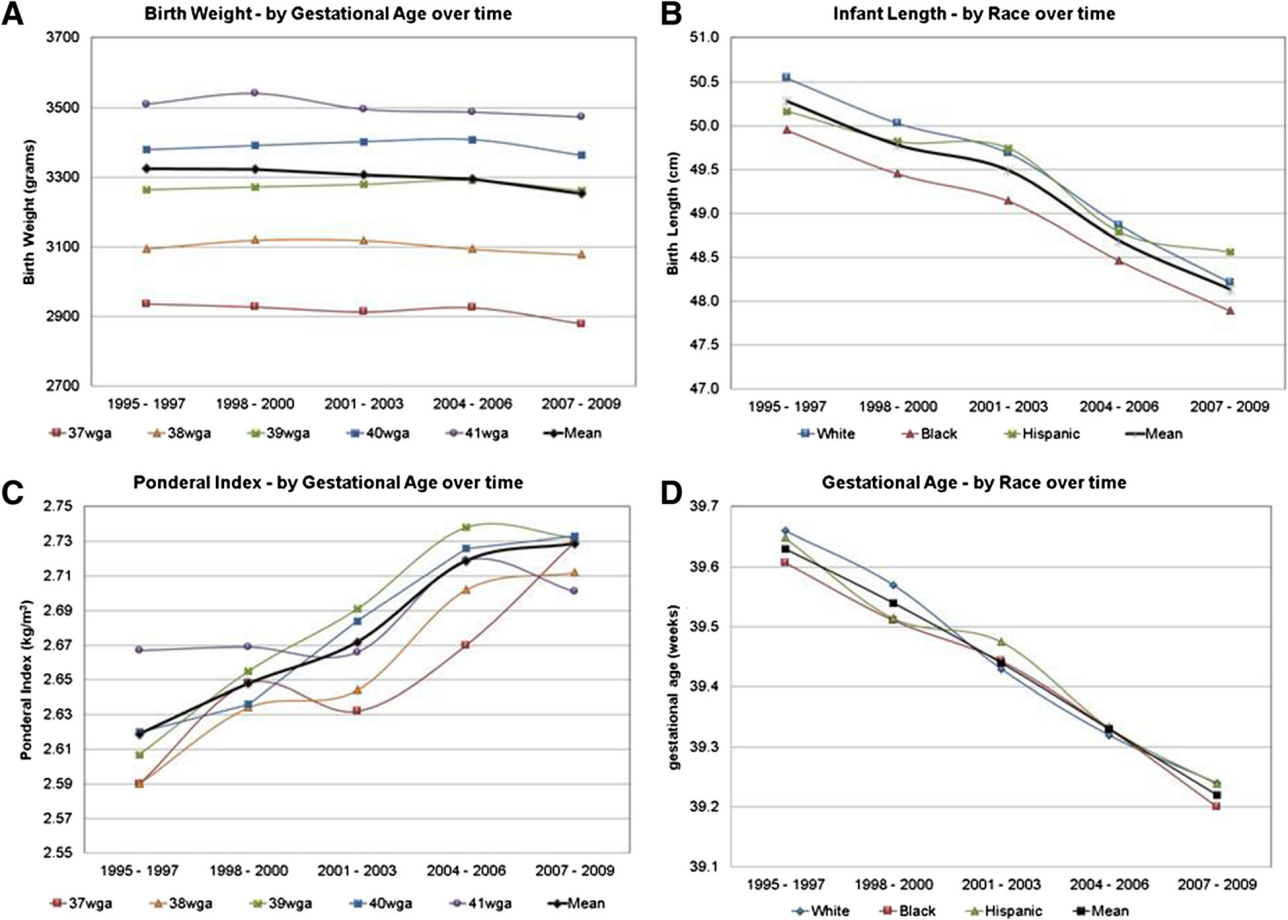
Change in Birth Outcomes over time. **a** Change in mean birth weight over time by gestational age. **b** Change in mean birth length over time. **c** Change in mean Ponderal Index over time. **d** Change in mean gestational age at delivery over time

A total of 43,217 singleton newborns were included into our final cohort. Between 1995 and January 1, 2010 mean BW for term singleton deliveries decreased by 72 g. Birth length decreased by 1.0 cm. This contributed to a significant increase over time in the neonatal Ponderal Index of 0.10 kg/m^3^ (Fig. 1a–c, Table 1).

**Table 1:** Demographic characteristics and outcomes over time

Variable	1995–1997 (*n* = 9447)	1998–2000 (*n* = 8801)	2001–2003 (*n* = 8399)	2004–2006 (*n* = 8418)	2007–2010 (*n* = 8182)	Total Change Over time	*P* value*
Maternal Age (years)	25.1 (6.2)	25.5 (6.2)	25.8 (6.3)	25.8 (6.2)	25.6 (6.2)	0.5	<0.001
Nulliparity (%)	39.4 %	36.5 %	37.8 %	38.6 %	38.8 %	−0.6 %	0.684
Smoking (%)	20.4 %	22.2 %	19.0 %	21.2 %	20.2 %	−0.2 %	0.383
Cesarean (%)	16.1 %	16.0 %	20.1 %	23.0 %	22.9 %	6.8 %	<0.001
BMI (kg/m2)	30.9 (6.6)	31.6 (6.7)	31.9 (6.9)	32.4 (7.2)	32.7 (7.3)	1.8	<0.001
Hypertension (%)	2.4 %	2.9 %	2.8 %	4.2 %	4.7 %	2.3 %	<0.001
Diabetes (%)	3.2 %	4.4 %	5.2 %	5.3 %	4.8 %	1.6 %	<0.001
%African American	37.2 %	38.3 %	38.1 %	42.1 %	45.4 %	8.2 %	<0.001
Mean BW (g)	3325 (483)	3324 (481)	3307 (475)	3295 (467)	3253 (457)	−72	<0.001
Median BW (g)	3310 [3010–3635]	3305 [3005–3630]	3295 [3995–3605]	3280 [2995–3590]	3240 [2955–3545]	−70	<0.0001
Infant Sex (%Male)	51.1 %	50.8 %	50.0 %	50.7 %	50.3 %	−0.8 %	0.283
Gestational Age (weeks)	39.6 (1.3)	39.5 (1.3)	39.4 (1.3)	39.3 (1.2)	39.2 (1.2)	−0.4	<0.001
Mean BL (cm)	50.4 (2.5)	50.1 (2.5)	50.0 (2.6)	49.6 (2.6)	49.4 (2.5)	−1.0	<0.001
Mean PI (kg/m^3^)	2.61 (0.33)	2.64 (0.32)	2.66 (0.34)	2.71 (0.36)	2.71 (0.35)	0.10	<0.001

Data presented as mean (standard deviation), median [interquartile range], or percentage; *BMI b*ody mass index, *BW* birth weight, *BL b*irth length, *PI* ponderal index

*P value listed in this table are from the Cochran-Armitage test for trend for binary variables (nulliparity, smoking, cesarean delivery, hypertension, diabetes mellitus, and infant sex) and linear regression analysis using contrasts to test for a linear trend for continuous variables (maternal age, BMI, percent African American, mean and median BW, gestational age, BL, and PI)

Table 1 also presents the changes in maternal and neonatal characteristics for the population over 15 years and in 3 year intervals. Maternal age increased over the 15-year period by 0.5 years. Self-reported race in the population changed from Caucasian to primarily African-American (11.47 % decrease in Caucasians and 8.18 % increase in African-Americans, Additional file 2: Figure S1A). The rate of hypertension and hypertensive disorders of pregnancy doubled to 4.67 %. Similarly, the prevalence of pre-existing diabetes mellitus also doubled to 2.07 % (Additional file 2: Figure S1B). Patients also became heavier over the study period with an increase in weight of 4 kg in their weight prior to delivery. Corresponding to the increase in maternal weight without significant change in height, the maternal body mass index (BMI) at delivery increased by a mean of 1.76 kg/m^2^. Additional file 2: Figure S1C shows this increase was consistent for all races, although African Americans had higher BMI’s than other ethnic groups. Parity remained stable with nearly one in three deliveries to a nulliparous woman. The rate of smoking was constant with 20 % of women using cigarettes of any amount throughout their pregnancy. Gestational age at delivery decreased by a mean of 0.4 weeks, a finding consistent amongst all races (Fig. 1d). Infant sex ratio remained constant (51.1–50.3 % male). The mode of delivery changed with an increase in primary cesarean deliveries from 9.99 to 11.92 % and a doubling in the rate of repeat cesareans from 5.25 to 11.05 %.

Because many factors that might affect birth weight changed over time within our cohort, we attempted to assess the contribution of the changes in maternal and newborn factors to the changes in BW, BL and PI using multiple regression. After assessing the correlation of each independent maternal and neonatal variable with BW, the clinically relevant and significant (*P* < 0.05) factors were included in the multiple regression model (Table 2). Similar regression analyses were performed for BL (Table 3) and PI (Table 4). In the multivariable regression model, after adjusting for potential confounders, we observed that every one unit increase in gestational age maternal BMI at delivery, and parity were associated with increases in birth weight. Male sex and pre-pregnancy diabetes were also associated with increases in birth weight in the regression model. Conversely, African American race, smoking, hypertension, and year of delivery were all associated with decreases in BW. Maternal smoking, African American race, year of delivery, and maternal hypertension were associated with a significant decrease in neonatal length while maternal BMI at delivery, parity, year of delivery, maternal diabetes, and gestational age were all associated with a significant increase in Ponderal Index.

**Table 2:** Estimated change in newborn birth weight by relevant maternal and newborn factors

--	r	Parameter Estimate	Standard Error	T-statistic	*P* Value
Gestational Age (weeks)	0.367	139.1	1.65	84.13	<0.0001
BMI (kg/m^2^)	0.198	13.5	0.3	44.86	<0.0001
African American Race	−0.15	−145.8	4.17	−34.93	<0.0001
Male Sex	0.13	122.9	4.03	30.44	<0.0001
Smoking	−0.134	−156.6	5.02	−31.21	<0.0001
Parity	0.101	98.9	4.21	23.51	<0.0001
Diabetes	0.074	168.9	9.95	16.97	<0.0001
Hypertension	−0.029	−75.5	11.34	−6.65	<0.0001
Year (per one year change)	−0.016	−1.7	0.48	−3.66	0.0003

Results from multiple regression model. Multivariate model adjusted R^2^ = 0.24

**Table 3:** Estimated change in newborn birth length by relevant maternal and newborn factors

--	r	Parameter Estimate	Standard Error	T-statistic	*P* Value
Gestational Age (weeks)	0.31	0.632	0.009	67.43	<0.0001
BMI (kg/m^2^)	0.091	0.033	0.002	19.50	<0.0001
African American Race	−0.108	−0.56	0.024	−23.69	<0.0001
Male Sex	0.154	0.786	0.023	34.36	<0.0001
Smoking	−0.115	−0.721	0.028	−25.36	<0.0001
Parity	0.000	0.003	0.024	0.13	0.9004
Diabetes	0.032	0.394	0.057	6.96	<0.0001
Hypertension	−0.018	−0.263	0.065	−4.06	<0.0001
Year (per one year change)	−0.103	−0.061	0.003	−22.72	<0.0001

Results from multiple regression model. Multivariate model adjusted R^2^ = 0.17

**Table 4:** Estimated change in newborn ponderal index by relevant maternal and newborn factors

--	r	Parameter Estimate	Standard Error	T-Statistic	*P* Value
Gestational Age (weeks)	0.046	0.013	0.001	9.27	<0.0001
BMI (kg/m^2^)	0.111	0.006	0	22.35	<0.0001
African American Race	−0.039	−0.028	0.003	−8.06	<0.0001
Male Sex	−0.041	−0.028	0.003	−8.56	<0.0001
Smoking	−0.013	−0.011	0.004	−2.73	0.006
Parity	0.109	0.077	0.003	22.43	<0.0001
Diabetes	0.044	0.073	0.008	8.94	<0.0001
Hypertension	−0.012	−0.022	0.009	−2.40	0.02
Year (per one year change)	0.114	0.009	0	23.44	<0.0001

Results from multiple regression model. Multivariate model adjusted R^2^ = 0.05; *BMI b*ody mass index

Further, the interaction between race and sex was explored in the analyses. Separate regressions were performed for each race and compared with the Chow test. While the test did find a significant difference between the races (*P* < 0.001), in examining the actual magnitude of estimates in the models, they are very similar (adjusted R^2^ = 0.24 for African American, adjusted R^2^ = 0.23 for all other races; Additional file 1: Table S3A–B).

## Discussion

Our data corroborate previous reports of a decrease in the term newborn birth weight over time. However, we also describe the significant decrease in birth length and resultant increase in Ponderal Index over time. Many of the changes in birth weight over time can be attributed to changes in obstetric practice and population demographics. Within our population a 72 g decrease in mean term birth weight was noted from 1995 through January 1, 2010. This was related to multiple factors including a decrease in gestational age at delivery, the increased percentage of African American patients, hypertension, and continued tobacco use.

Our study has several strengths. We used a large number of patients from a well-established, prospectively collected database. The patient population is similar to many inner-city hospitals, thus the results could be generalized to similar populations, but may not be applicable to others. This is one of the first studies of an entire population to report on changes in birth length. The multiple regression controlled for common confounding variables. The methods of measuring neonatal weight and length at birth did not change over the study period. To explore the accuracy of the clinical birth lengths, we analyzed the clinical measure of neonatal length in 450 infants who were also subjects in an anthropomorphic study that included length at birth by a trained research nurse using a measuring board. The measurements correlated well with an r = 0.67 (*P* < 0.001, unpublished) which did not significantly change over time.

This analysis also has limitations. It was retrospective and limited by what data was available and confined to a single site. The patient population is not representative of the overall population in the United States. Some potential confounders, such as maternal pregravid BMI, gestational weight gain, glycemic control, degree of hypertension, gestational age at first ultrasound, socio-economic status, and paternal height and weight could not be evaluated. As birth weight is dependent on these and other, unaccounted for factors, the r^2^ values for multivariate analyses for BW, BL, and PI were each relatively low (0.05–0.24). Thus, while the variables entered into our model, including year, were significantly correlated, many other factors as described above may also affect the changes seen in BW over time.

Several previous studies have reported the change in BW over time in large populations. In 2010 Donahue et al. reported a 52 g decrease in mean newborn BW amongst 36,827,828 deliveries over 15 years in a national birth certificate database [13]. Similar to our findings, the decrease remained significant after controlling for gestational age at delivery, maternal age, race, parity, smoking, weight gain, and medical comorbidities. Zhang et al. found a significant association between the increasing use of labor inductions and the decline in gestational age and BW, proposing that the change in BW was due more to decreases in gestational age from alterations in obstetric practice rather than neonatal biometric changes [14]. Schiessel et al. assessed 695,707 mother-infant pairs in Bavaria and found an increase in maternal weight gain yet a decrease in BW of nineteen grams [15]. These results are consistent with our finding of increasing maternal BMI at delivery, but the Schiessel study did not adjust for the decrease in gestational age. As term fetuses gain approximately 10–20 g/day [32], changes in gestational age may have accounted for the some of the change in mean birth weight. Morisaki et al. reported that in a population where length of gestation at term did not change, birth weight and fetal growth declined. Differences geography, ethnicity of the study population, and the prevalence of overweight and obesity may explain some of these differences in Morisaki’s results and our findings [34].

The majority of the decrease in birth weight over time was related to concomitant changes in maternal demographics, especially race and comorbidities, and gestational age. This suggests that the single parameter of birth weight alone may be an insufficient assessment of intrauterine growth. Our study is among the first to consider neonatal length and the Ponderal Index in our evaluating trends in fetal growth. The trend of a decrease in birth weight and length is clinically important insomuch as it represents a change in intrauterine growth which has potential long-term implications for both child and adult health, relative to fetal programming and increasing adiposity.

The concept of decreased neonatal length has also been referred to as stunting or decreased crown-heel length for gestational age [35]. Stunting has primarily been related to inadequate nutrition during pregnancy and the first years of life [36]. However, based on our findings of a 4.2 % decrease in length and increases in maternal metabolic syndrome of pregnancy, i.e., hypertension, diabetes, and BMI at delivery, we may be observing the effects of the broader definition of malnutrition, i.e., a diet poor in healthy nutrients but with a surfeit of poor quality calories. Being born short increases the risk of adult shortness [37] and related problems and may be a surrogate for lean body mass. The ratio of fat to lean body mass may be a better predictor of later metabolic disease than either anthropometric measure alone.

How useful is the concept of Ponderal Index in assessing fetal growth? Rohrer first proposed the term in 1921 as the “corpulence index” used to evaluate growth in neonates and children after World War I in Germany (33The term is useful because mass/volume (length^3^) is the definition of density used in many formulations of body composition. In a sample of 215 Caucasian, African American and Hispanic neonates, there was a significant correlation between Ponderal Index and percent body fat using air densitometry or Pea Pod (R =0.40, *P* = <0.001, see Additional file 3: Figure S2). Hence the concern is that the decrease in birth weight relating to demographic and practice patterns may be more than offset by other factors affecting fetal length and possibly lean body mass relative to long-term health of the neonate. The change in Ponderal Index from 1995 to 1997 to 2007–2009 (2.61 to2.71) represents approximately a 10 % increase in Ponderal Index over time.

## Conclusions

In summary, consistent with recent reports we found a decrease in mean birth weight over time. The majority of this change can be attributed to variations in demographic factors and obstetrical practice affecting fetal growth. We also noted a significant decrease in length at birth and increase in Ponderal Index over time. These data stress the importance of considering factors beyond birth weight alone in the assessment of fetal growth. The increases in maternal BMI coupled with the increased Ponderal Index as an estimate of neonatal adiposity are consistent with the concept of developmental programming relating to the increase in the worldwide obesity epidemic.

## Additional files

Additional file 1: Table S1. Univariate Pearson correlation coefficients between independent variables and birth weight, birth length, and ponderal index. **Table S2.** Univariate Pearson correlation coefficients between independent variables to assess for multicollinearity. **Table S3A.** Estimated Change in Newborn Birth Weight by Relevant Maternal and Newborn Factors by Race for African Americans. **Table S3B.** Estimated Change in Newborn Birth Weight by Relevant Maternal and Newborn Factors by Race for non-African Americans. (DOC 64 kb) Additional file 2: Figure S1. Change in maternal demographics over time. A Change in maternal race over time B Change in Maternal comorbid conditions of HTN and DM over time. C Change in mean Maternal Body Mass Index over time. (JPEG 55 kb) Additional file 3: Figure S2. Correlation between Ponderal Index and percent of neonatal body fat as measured by the PeaPod. (JPEG 52 kb)

## Abbreviations

AGA: Average for gestational age
BMI: Body mass index
BW: Birth weight
LGA: Large for gestational age
PI: Ponderal index
SGA: Small for gestational age
