# Supplementary material for: A retrospective cohort study of factors relating to the longitudinal change in birth weight
Source: BMC Pregnancy Childbirth. 2015 Dec 22;15:344. doi: 10.1186/s12884-015-0777-8 (PMC4687143; doi:10.1186/s12884-015-0777-8)
Supplement: Additional file 1: Table S1. — Univariate Pearson correlation coefficients between independent variables and birth weight, birth length, and ponderal index. Table S2. Univariate Pearson correlation coefficients between independent variables to assess for multicollinearity. Table S3A. Estimated Change in Newborn Birth Weight by Relevant Maternal and Newborn Factors by Race for African Americans. Table S3B. Estimated Change in Newborn Birth Weight by Relevant Maternal and Newborn Factors by Race for non-African Americans. (DOC 64 kb) [file 12884_2015_777_MOESM1_ESM.doc]

**Additional file 1 Table S1** Univariate Pearson correlation coefficients between independent variables and birth weight, birth length, and ponderal index

|  | BW (g) | BL (cm) | PI (kg/m^3^) |
| --- | --- | --- | --- |
| Gestational Age (weeks) | 0.366 | 0.326 | 0.024 |
| Parity | 0.071 | -0.031 | 0.112 |
| BMI (kg/m^2^) | 0.215 | 0.090 | 0.132 |
| African American Race | -0.122 | -0.101 | -0.016 |
| Male sex | 0.129 | 0.152 | -0.040 |
| Smoking | -0.130 | -0.118 | -0.006 |
| Diabetes | 0.064 | 0.006 | 0.064 |
| Hypertension | -0.042 | -0.034 | -0.007 |
| Year | -0.050 | -0.141 | 0.0120 |

BMI (Body Mass Index); BW (Birth Weight); BL (Birth Length); PI (Ponderal Index)

**Additional file 1 Table S2** Univariate Pearson correlation coefficients between independent variables to assess for multicollinearity

|  | Gestational Age (weeks) | Parity | BMI (kg/m^2^) | African American Race | Male sex | Smoking | Diabetes | Hypertension | Year |
| --- | --- | --- | --- | --- | --- | --- | --- | --- | --- |
| Gestational Age (weeks) | 1 | 0.081 | 0.046 | -0.019 | -0.012 | -0.025 | -0.133 | -0.072 | -0.120 |
| Parity | -0.081 | 1 | 0.062 | 0.030 | 0.002 | 0.095 | 0.027 | -0.066 | 0 |
| BMI (kg/m^2^) | 0.046 | 0.062 | 1 | 0.134 | 0.011 | -0.036 | 0.163 | 0.101 | 0.091 |
| African American Race | -0.019 | 0.030 | 0.134 | 1 | -0.002 | -0.073 | -0.015 | 0.028 | 0.059 |
| Male sex | -0.012 | 0.002 | 0.011 | -0.002 | 1 | -0.002 | 0.007 | 0.007 | -0.004 |
| Smoking | -0.025 | 0.095 | -0.036 | -0.073 | -0.002 | 1 | -0.015 | -0.029 | -0.004 |
| Diabetes | -0.133 | 0.027 | 0.163 | -0.015 | 0.007 | -0.015 | 1 | 0.025 | 0.032 |
| Hypertension | -0.072 | -0.066 | 0.101 | 0.028 | 0.007 | -0.029 | 0.025 | 1 | 0.047 |
| Year | -0.120 | 0 | 0.091 | 0.059 | -0.004 | -0.004 | 0.032 | 0.047 | 1 |

BMI (Body Mass Index)

**Additional file 1: Table S3A** Estimated Change in Newborn Birth Weight by Relevant Maternal and Newborn Factors by Race for African Americans

| -- | r | Parameter Estimate | Standard Error | T-Statistic | P - Value |
| --- | --- | --- | --- | --- | --- |
| Gestational Age (weeks) | 0.378 | 137.1 | 2.51 | 54.67 | <0.001 |
| BMI (kg/m^2^) | 0.201 | 12.3 | 0.43 | 28.84 | <0.001 |
| Male Sex | 0.124 | 114.1 | 6.17 | 18.49 | <0.001 |
| Smoking | -0.114 | -139.3 | 8.32 | -16.74 | <0.001 |
| Parity | 0.088 | 83.4 | 6.56 | 12.71 | <0.001 |
| Diabetes | 0.102 | 234.7 | 15.84 | 14.8 | <0.001 |
| Hypertension | -0.038 | -88.6 | 16.05 | -5.52 | <0.001 |
| Year (per one year change) | -0.012 | -1.3 | 0.72 | -1.83 | 0.067 |

Legend: Results from multiple regression model. Multivariate model adjusted R^2^ =0.24; BMI (Body Mass Index)

**Additional file 1: Table S3B** Estimated Change in Newborn Birth Weight by Relevant Maternal and Newborn Factors by Race for non-African Americans

| -- | r | Parameter Estimate | Standard Error | T-Statistic | P - Value |
| --- | --- | --- | --- | --- | --- |
| Gestational Age (weeks) | 0.366 | 140.7 | 2.19 | 64.24 | <0.001 |
| BMI (kg/m^2^) | 0.198 | 14.7 | 0.42 | 34.66 | <0.001 |
| Male Sex | 0.134 | 128.3 | 5.32 | 24.12 | <0.001 |
| Smoking | -0.147 | -165.8 | 6.31 | -26.28 | <0.001 |
| Parity | 0.111 | 108.8 | 5.49 | 19.83 | <0.001 |
| Diabetes | 0.057 | 129.0 | 12.79 | 10.09 | <0.001 |
| Hypertension | -0.022 | -63.9 | 15.88 | -4.02 | <0.001 |
| Year (per one year change) | -0.018 | -2.07 | 0.63 | -3.27 | 0.001 |

Legend: Results from multiple regression model. Multivariate model adjusted R^2^ =0.23
