# Supplementary figures and images for: A retrospective cohort study of factors relating to the longitudinal change in birth weight
Source: BMC Pregnancy Childbirth. 2015 Dec 22;15:344. doi: 10.1186/s12884-015-0777-8 (PMC4687143; doi:10.1186/s12884-015-0777-8)

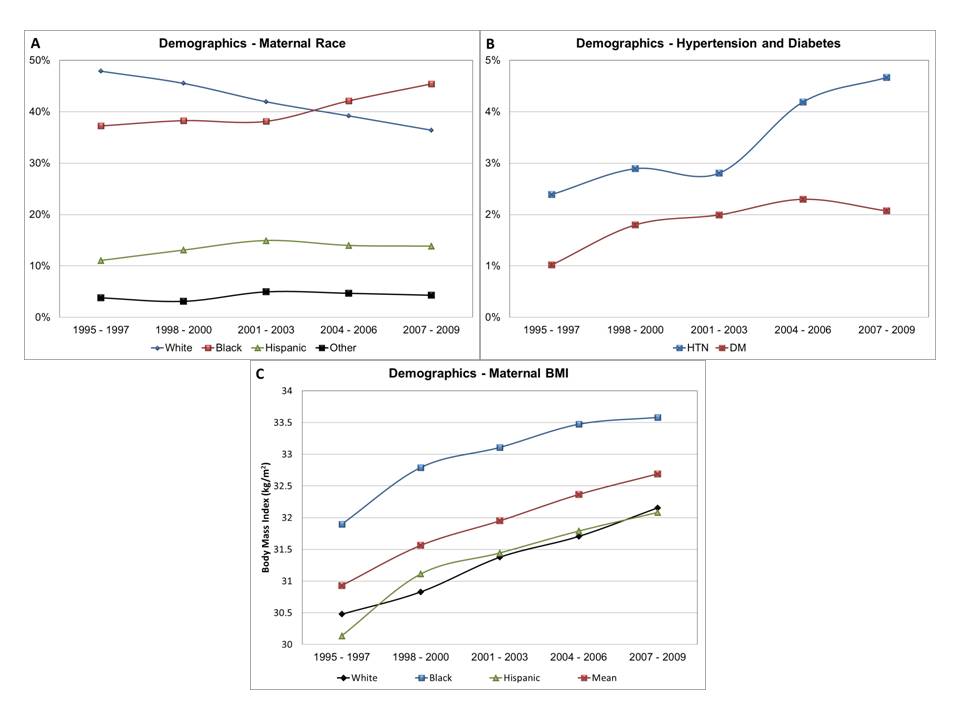

Supplement: Additional file 2: Figure S1. — Change in maternal demographics over time. A Change in maternal race over time B Change in Maternal comorbid conditions of HTN and DM over time. C Change in mean Maternal Body Mass Index over time. (JPEG 55 kb) [file 12884_2015_777_MOESM2_ESM.jpeg]

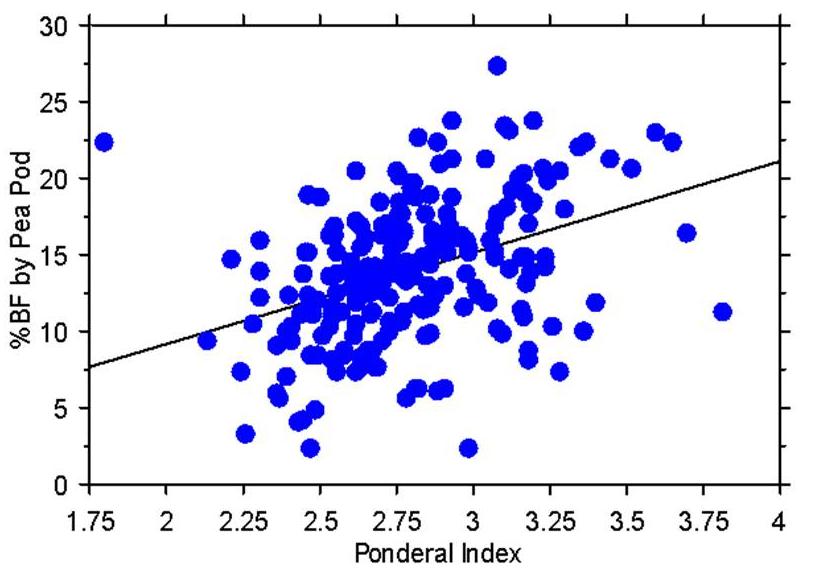

Supplement: Additional file 3: Figure S2. — Correlation between Ponderal Index and percent of neonatal body fat as measured by the PeaPod. (JPEG 52 kb) [file 12884_2015_777_MOESM3_ESM.jpeg]
